# Prototypic Lightweight Alloy Design for Stellar‐Radiation Environments

**DOI:** 10.1002/advs.202002397

**Published:** 2020-09-30

**Authors:** Matheus A. Tunes, Lukas Stemper, Graeme Greaves, Peter J. Uggowitzer, Stefan Pogatscher

**Affiliations:** ^1^ Chair of Nonferrous Metallurgy Montanuniversitaet Leoben Leoben A‐8700 Austria; ^2^ Christian Doppler Laboratory for Advanced Aluminium Alloys Chair of Nonferrous Metallurgy Montanuniversitaet Leoben Leoben A‐8700 Austria; ^3^ School of Computing and Engineering University of Huddersfield Huddersfield HD1 3DH United Kingdom; ^4^ Laboratory of Metal Physics and Technology Department of Materials ETH Zürich Zürich 8093 Switzerland

**Keywords:** alloy design, aluminium alloys, extreme environments, space exploration, space materials

## Abstract

The existing literature data shows that conventional aluminium alloys may not be suitable for use in stellar‐radiation environments as their hardening phases are prone to dissolve upon exposure to energetic irradiation, resulting in alloy softening which may reduce the lifetime of such materials impairing future human‐based space missions. The innovative methodology of crossover alloying is herein used to synthesize an aluminium alloy with a radiation resistant hardening phase. This alloy—a crossover of 5xxx and 7xxx series Al‐alloys—is subjected to extreme heavy ion irradiations in situ within a TEM up to a dose of 1 dpa and major experimental observations are made: the Mg_32_(Zn,Al)_49_ hardening precipitates (denoted as T‐phase) for this alloy system surprisingly survive the extreme irradiation conditions, no cavities are found to nucleate and displacement damage is observed to develop in the form of black‐spots. This discovery indicates that a high phase fraction of hardening precipitates is a crucial parameter for achieving superior radiation tolerance. Based on such observations, this current work sets new guidelines for the design of metallic alloys for space exploration.

## Graphical abstract

Heavy ion irradiation is used to assess the real‐time irradiation response of a new aluminium alloy under conditions that extrapolates the radiation dose levels within the solar system. The hardening phase ‐ Mg_32_(Zn,Al)_49_ ‐ is found to be radiation tolerant and new guidelines for the design of space materials are proposed.

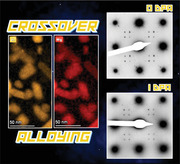

## 1 Introduction

Unveiling the unknown is an inherent desire of civilization. Since the first human‐made object successfully completed several orbits around the planet Earth—the soviet satellite Sputnik in 1957^[^ ^1^ ^]^—space exploration remains, a topic of great interest not only for scientists, but for the general public. To successfully exploit space exploration a range of hurdles must be overcome, with most of them being in the area of materials science. In the past 80 years, this branch of science has significantly evolved to better withstand the extreme conditions typically found in the space environment.

With a new era of space exploration in part being driven by the private sector,^[^ ^2^, ^3^ ^]^ it is expected that space exploration will grow over the next years as a potential business model (e.g., space tourism), thus emphasising the need for new materials and alloys for satellites, spacecrafts, spaceprobes, and derivatives. In addition to this demand, some spacefaring powers are still deeply engaged in the development of their civilian and military space programmes.^[^ ^4^ ^]^ Undoubtedly, the capability of a material to hold its designed properties while exposed to the degradation mechanisms found in space is one of the major challenges for the development of future space materials.^[^ ^5^ ^]^

### 1.1 Space Materials Requirements

Materials degradation mechanisms in space materials were recently reviewed considering the state‐of‐the‐art of spacecraft materials.^[^ ^5^, ^6^ ^]^ These degradation mechanisms may operate in a synergistic manner leading to catastrophic failure when not properly addressed, thus increasing the challenge in the design and selection stages.

Within the multiple aspects of the extreme conditions present in the space environment, materials can be subjected to vacuum conditions (pressures around 10${}^{-4}$ Pa) that may lead materials to outgas that can result in gas‐related problems (corrosion, oxidation, and embrittlement) and contamination.^[^ ^7^ ^]^ Thermal cycling is another concern when a material is exposed to high (stellar light) and low temperatures (shadowing conditions). Abrupt thermal variations can cause cracking, delamination of coatings, mechanical performance deterioration including severe thermal expansion and contraction issues.^[^ ^8^, ^9^ ^]^

Ultraviolet radiation‐induced ionization of upper atmosphere molecules is reported to generate active monoatomic species (ionized plasma) such as O which can react with polymer‐based materials containing C, N, S, and H leading to severe degradation via chemical reactions and erosion.^[^ ^5^ ^]^ Active monoatomic species can also cause significant embrittlement of metallic alloys.^[^ ^10^, ^11^, ^12^, ^13^ ^]^ Functional coatings can be applied to spacecraft materials in order to reduce the degradation yield, but under these conditions, such degradation (severe in some cases) has been reported in a series of materials including hard ceramic and metallic thin films.^[^ ^14^ ^]^

The high‐speed impact of micrometeoroids and space debris is another major concern for the design of space materials. Reports show that the formation of a near‐earth ring of debris has already caused damage to satellites and also the international space station,^[^ ^15^, ^16^ ^]^ thus a strong mechanical shield is required on spacecrafts in order to minimize the impact of these collisions. Another aspect in this context is that damaged parts should be easily manufactured and replaceable, thus limiting the spectrum of materials selection.

In summary, the following list of requirements must be observed in selecting space materials^[^ ^5^, ^6^, ^7^, ^8^, ^9^, ^10^, ^11^, ^12^, ^13^, ^14^, ^15^, ^16^, ^17^ ^]^:

– Strength‐to‐weight ratio: space materials require high mechanical performance with reduced weight. High‐strength lightweight materials are the optimal case.
– Thermal performance: materials to cope with high thermal gradients while holding excellent performance. Tribology of coatings must also address the space environment.
– Corrosion protection: mainly against active monoatomic species arising from ionized plasma.
– The triad of manufacturability, repairability, and cost: space materials should be easily machinable, repairable and replaceable always considering the costs associated with such processes.

The space environment has high levels of energetic charged particle radiation, therefore radiation tolerance is also important. The following subsection will briefly introduce the harmful and extreme radiation levels in space, a field known as “space weather.”^[^ ^18^ ^]^

### 1.2 Brief Introduction to Space Weather

Space weather investigates the relationship between temporal variations of the Sun's activity (solar cycles) in terms of its winds and the surrounding environment of the planet Earth, mainly in terms of its magnetosphere.^[^ ^18^, ^19^, ^20^, ^21^, ^22^, ^23^, ^24^, ^25^, ^26^ ^]^ Solar wind is essentially composed of energetic irradiation in the form of galactic cosmic rays (GCR) or solar energetic particles (SEP).^[^ ^21^, ^22^, ^27^ ^]^ As most of the radiation damage yield within the solar system has its origins in the Sun, it will be herein referred to as stellar‐radiation.

Under the normal conditions of the solar cycles, a great fraction of stellar‐radiation reaching Earth is shielded by the Earth's magnetic field creating the Van Allen (VA) radiation belts.^[^ ^28^ ^]^ Considering the radius of Earth (denoted as $R_{E}$), the inner VA belt ranges from 1.2 to 3$R_{E}$ while the outer VA belt extends from 3 to 7$R_{E}$.^[^ ^29^ ^]^ Within the mentioned ranges most of GCRs and SEPs are trapped by Earth's magnetic field, therefore offering some important natural shielding protection where human‐based space activity generally takes place.

Under abnormal conditions where the solar cycles are disrupted by events such as coronal mass ejections (CMEs) and solar flares (SFs), the flux and energy of SEPs can increase markedly above the normal levels. Measurements taken by the solar dynamics observatory (NASA/SDO) in September 2017^[^ ^27^, ^30^, ^31^ ^]^ revealed the occurrence of large SF events where SEPs in the form of highly energetic proton beams were detected to reach Earth with energies in the range of 2.5 to 433.0 MeV.^[^ ^27^ ^]^ The dose levels were also observed to peak during these events as measured by the cosmic ray telescope for the effects of radiation (CRaTER) orbiting the Moon aboard the lunar reconnaissance orbiter (LRO).^[^ ^27^, ^32^ ^]^ For example, Schwadron et al. reported that between the 10th and 14th of September 2017, the dose rate measured by CRaTER was around 1 Gy·day${}^{-1}$ which can cause immediate symptoms of acute radiation syndrome in the human body.^[^ ^33^, ^34^, ^35^, ^36^ ^]^

Therefore, it must be considered that within the solar system, the deleterious effects of stellar‐radiation in materials and also in crew members may impair future plans for space exploration. This is particularly important for human‐based exploration outside the VA belts and under abnormal solar weather. Future space materials will thus also require the ability of resisting atomic collisions while serving as a shield to this irradiation.

**Figure 1:**
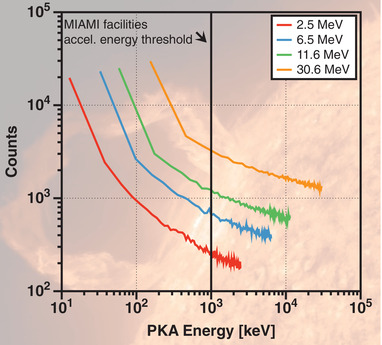
PKA energies calculated using SRIM‐2013Pro considering a pure Al target with thickness of 1 cm. Incident proton energies of 2.5, 6.5, 11.6, and 30.6 MeV were used and they correspond to the most common energies measured during the solar flare events from September 2017 as reported by Schwadron et al.^[^ ^27^ ^]^ The dashed vertical line in the centre of the plot represents the maximum achievable acceleration energy in the MIAMI facilities.^[^ ^37^ ^]^ Most of SEPs emitted during CMEs events generate radiation damage within the achievable energy range for particle accelerators currently in‐use for nuclear materials research. Note: in the y‐axis, counts is the discrete number of PKAs.

Given the complex multidisciplinary challenge, space materials research can benefit from almost a century of accumulated knowledge in the field of radiation damage in nuclear materials. Using the most common proton energies as measured in the SFs events from September 2017,^[^ ^27^ ^]^ the energy distribution of primary knocked‐on atoms (PKAs) in pure Al (already in‐use as a space material^[^ ^38^, ^39^, ^40^, ^41^, ^42^, ^43^, ^44^, ^45^, ^46^ ^]^) was calculated with the SRIM‐2013Pro Monte Carlo code.^[^ ^47^ ^]^ **Figure** 1 shows that while the SFs proton energies are as high as 30.6 MeV (orange line), the great majority of PKA energies are less than 1 MeV (for 1 cm of Al as a target). This suggests that the current methodology for the study of radiation damage in nuclear materials with low‐ and medium‐energy particle accelerators can be also used for the future investigation of SPE effects in space materials. It is worth emphasising that the measured proton fluxes decrease significantly with increasing energy as reported by Schwadron et al.^[^ ^27^ ^]^ This emphasises that most of the radiation damage yield is due to collisions of SF protons with energies of around 2.5 MeV and the subsequent PKAs energy distribution will be hundreds of keV.

### 1.3 Overview of Irradiation Response of Commercial Al‐Based Alloys

Due to its light‐weight and suitable properties for space applications, one can consider Al and its alloys as a strategic class of materials for application in space, but radiation damage still remains a major challenge in these alloys. A comprehensive, but limited bibliography on the neutron irradiation response of Al‐based alloys reports mainly on the 5xxx (AlMg) and 6xxx (AlMgSi) series of alloys.^[^ ^48^, ^49^, ^50^, ^51^, ^52^, ^53^, ^54^, ^55^, ^56^, ^57^ ^]^ A recent review was presented by Kolluri.^[^ ^58^ ^]^ These commercial Al‐based alloys are still in‐service worldwide as nuclear fuel cladding alloys in nuclear research reactors where the normal operation temperature is limited to around 293 K.^[^ ^56^ ^]^ Four major effects of neutron irradiation on Al‐based alloys were identified from the literature^[^ ^48^, ^49^, ^50^, ^51^, ^52^, ^53^, ^54^, ^56^, ^57^, ^58^ ^]^ and can be summarized as follows:

i) Trapping of irradiation‐induced interstitial solutes point defects in excess: point defects can trap interstitial solutes in age‐hardenable alloys which results in the retardation of the radiation‐induced segregation (RIS) and precipitation (RIP) mechanisms.^[^ ^50^ ^]^ Although such retardation has been similarly reported in age‐hardenable Fe‐C alloys,^[^ ^59^, ^60^ ^]^ this phenomenon is opposed to common reports on austenitic stainless steels under energetic irradiation environments.^[^ ^61^, ^62^, ^63^, ^64^ ^]^ Recent values of solute‐vacancy binding energies were calculated by Wolverton;^[^ ^65^ ^]^
ii) Guinier‐Preston zones (GPZs) and precipitation kinetics acceleration: as a result of neutron‐induced activation and transmutation of ^27^AI, ^28^Si via the nuclear reaction shown in Equation (1),^[^ ^66^ ^]^ increased nucleation and growth rates of GPZs and precipitation of phases such as Mg${}_{2}$Si were observed in commercial AlMg and AlMgSi alloys at temperatures where vacancies are mobile.^[^ ^50^, ^53^ ^]^ Acceleration of $\theta^{\prime }$‐phase (Al${}_{2}$Cu) precipitation in Al‐based Cu containing alloys was also reported under low temperature neutron irradiation.^[^ ^48^, ^49^ ^]^ As a consequence, severe embrittlement (i.e., loss of ductility) of the Al‐based alloy is reported.^[^ ^48^, ^49^, ^50^, ^51^ ^]^
  (1) $${}^{27}\text{Al}{\left(n,\gamma \right)}^{28}\text{Al}\to^{28}\text{Si}+\beta^{-}$$
iii) Radiation‐induced defects and heterogeneous nucleation: the formation and growth of extended radiation damage defects (e.g. black‐spots, dislocation loops and voids) can serve as preferential sites for heterogeneous nucleation of either GPZs or hardening phases. It is worth emphasising that the saturation of displacement damage defects can readily occur in the Al matrix as solid‐state diffusion is accelerated even at low temperatures.^[^ ^67^, ^68^, ^69^, ^70^ ^]^
iv) Morphological changes in the hardening phases: attributed to thermal spikes and large defect cascades,^[^ ^71^, ^72^ ^]^ the hardening phases of Al‐based alloys are reported to either breakup or dissolve under irradiation.^[^ ^50^, ^54^ ^]^ Radiation softening was also reported. Using tensile testing, Ismail measured that after neutron irradiation at 323 K, commercial AlMgSi alloys were softer as a result of the irradiation‐induced dissolution of age‐hardening precipitates^[^ ^54^ ^]^; the degree of irradiation softening was superior for the alloy with higher initial hardness.

Investigations into the response of Al‐based alloys to ion irradiation are not as common as for neutron irradiation. Lohmann et al. investigated the effects of high‐energy protons (600–800 MeV) on both microstructure and elasto‐plastic behavior of commercial AlMg and AlMgSi alloys^[^ ^73^ ^]^ up to a maximum dose of 0.2 dpa at lower temperatures (≤ 373 K). The alloys were subjected to irradiation in both annealed (O and T6) and cold‐worked states. It was reported that the hardening phases (Mg${}_{2}$Si known as β‐phase^[^ ^74^ ^]^) were dissolved during irradiation and electron‐microscopy evidence (only BFTEM micrographs) was presented as opposed to the neutron irradiation study from Ismail.^[^ ^54^ ^]^ Lohmann et al.^[^ ^73^ ^]^ also reported that post‐irradiation tensile experiments revealed considerable radiation‐induced softening and loss‐of‐strength at lower doses. In addition, after irradiation, RIP was not detected in the fully annealed (i.e., AA6061‐O) specimen.

### 1.4 Main Objectives of This Present Work

Using the recently introduced lightweight alloy design strategy known as “crossover alloying,”^[^ ^75^, ^76^, ^77^, ^78^ ^]^ the metallurgical merging of the beneficial properties from 5xxx (AlMg) with the 7xxx (AlZn) alloy series such as high formability and high strength, respectively, this paper is aimed at investigating the heavy ion irradiation response of a distinct crossover alloy herein developed: the overaged AlMg_4.7_ Zn_3.4_ (in wt%). Age‐hardening for this alloy was achieved through the controlled and finely dispersed precipitation of the T‐phase—Mg_32_(Zn,Al)_49_^[^ ^79^, ^80^ ^]^ —which consists of an intermetallic superstructure.

Given the importance of Al and its lightweight alloys to the space materials industry and the fact they can (or be engineered to) satisfy the spacecraft materials requirements as exhibited in Section 1.1, heavy ion irradiation was selected to more closely emulate (under prototypic conditions) the Al‐PKA recoil spectrum of the most common high energy protons that are emitted during SF events as described in the space weather Section 1.2. As can be concluded from the state‐of‐the‐art on radiation response of commercial Al‐based alloys (reviewed in Section 1.3), their radiation resistance is mainly affected by the response of the hardening phases. Therefore, this work focuses on a detailed electron‐microscopy characterization of the T‐phase precipitates before, during and after heavy ion irradiation.

## 2 Experimental Section

### 2.1 Alloy Synthesis, Processing, and Electron‐Microscopy Sample Preparation

The alloy (≈ 100 g) was melted in a graphite crucible and shed into a copper mold using a laboratory scale vacuum induction furnace (Indutherm MC100V). The slab was scalped and the surface treated by milling. After pre‐heating for 1 h at 738 K in an air circulating furnace, the sample was hot rolled to a thickness of 4 mm and subsequently cold rolled in order to generate a final cold rolling degree of 50%. The cold rolled sheet was then solution heat‐treated at 738 K for 35 min followed by water quenching to achieve a supersaturated solid solution. After the solution heat‐treatment, two‐step artificial aging‐3 h at 373 K + 16 days at 448 K – was performed in a circulating oil bath. This heat‐treatment condition is herein referred to as overaged. For thin foil preparation, 3 mm disks were punched out of a 10 mm × 10 mm sample, which was cut from the centre of the heat treated sheet and had been ground and polished to a thickness of around 100 µm. The measured elemental composition of the alloy in its final state is shown in **Table** 1. It is worth emphasising that this crossover alloy is based on EN AW‐5182 alloy, but modified by adding Zn in order to achieve age hardenability.

**Table 1:** Elemental composition of the studied crossover AlMgZn alloy

Alloying Elements	Si	Mg	Mn	Fe	Cr	Zn	Cu	Res.	Al
Composition [wt%]	0.1	4.7	0.4	0.2	0.1	3.4	<0.1	<0.1	Balance

Electrochemical jet polishing (ECJP) was used to prepare electron‐transparent samples for the scanning transmission electron microscope (STEM) and also for ion irradiationin situ within a TEM. ECJP was performed on the punched 3 mm disks using a Struers Tenupol‐5 and the electrolyte used was a solution of 33% nitric acid with 66% methanol (in vol%). The temperature of the electrolyte during the ECJP was constant at 263 K and the voltage applied was set to 12 V.

### 2.2 Heavy Ion Irradiation In Situ Within a TEM

**Figure 2:**
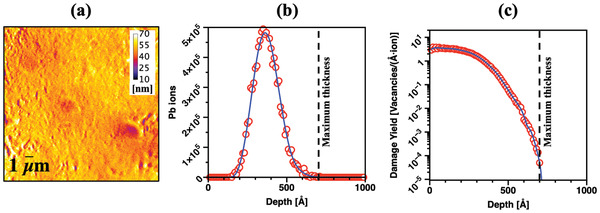
Experimental parameters for 100 keV Pb${}^{+}$ ion irradiation: a) EFTEM thickness map of the studied alloy, b) the implantation yield of Pb ions, and c) damage yield as a function of the thickness of the specimen.

Electron‐transparent samples with an average thickness between 50 and 70 nm —measured by energy‐filtered TEM (EFTEM) as shown in **Figure** 2a)—were subjected to heavy ion irradiation *in situ* within a Hitachi H9500 TEM at the MIAMI‐2 facility at the University of Huddersfield. Complete details of the experimental apparatus can be found elsewhere.^[^ ^37^ ^]^ In the experiments reported in this paper, a 100 keV Pb${}^{+}$ ion beam collided with the samples at an angle of 18.7${}^{\circ }$ with respect to the electron beam which is normal to the sample. No additional heating was applied to the specimen and the irradiations were carried out at room temperature (293 K). During the experiments, no rise in the temperature was detected. The Pb implantation profile and the damage yield for such an ion beam are shown in Figure 2b,c, respectively. For this ion irradiation set‐up, each Pb ion collision with the alloy matrix generated ≈ 10${}^{3}$ vacancies as calculated with the Monte Carlo code SRIM‐2013Pro^[^ ^47^ ^]^ and by using a procedure suggested by Stoller et al.^[^ ^81^ ^]^ The ion flux measured at the specimen position was $5.10\times 10^{11}$ $\mathrm{ions}\cdot {\mathrm{cm}}^{-2}\cdot s^{-1}$ and the samples were irradiated up to a fluence of $4.28\times 10^{14}\mathrm{ions}\cdot {\mathrm{cm}}^{-2}$ corresponding – in the Al matrix – to 1 displacement‐per‐atom (dpa) averaged over the specimen thickness (assumed to be 70 nm for the fluence‐to‐dpa calculations).

### 2.3 Pre and Postirradiation Characterization

Pre and postirradiation characterization of the electron‐transparent samples was performed in a Thermo Fisher Talos F200X S/TEM operating a Schottky field emission gun at 200 keV and located at the Montanuniversitaet Leoben. The STEM was equipped with SuperX detectors for elemental mapping using Energy Dispersive X‐ray (EDX) spectroscopy. STEM micrographs using the high‐angle annular dark‐field (HAADF) and bright‐field (BF‐STEM) detectors were acquired with a camera length of 98 mm. This microscope was also used in the TEM mode for high‐resolution (HRTEM) screening of the samples.

### 2.4 Statistical Analysis

Statistics has been used in this work to quantify the area of the T‐phase precipitates before and after irradiation. The Mg‐maps obtained after STEM–EDX screening of different sampling areas before and after irradiation were used to carry out particle size analysis with the ImageJ software following procedures described in the literature (histogram‐based segmentation and thresholding).^[^ ^82^ ^]^

## 3 Results

For clarification, this section has been subdivided into three subsections in order to report on the characterization carried out on the crossover AlMgZn alloy before, during and after irradiation.

### 3.1 Alloy Preirradiation Characterization

**Figure 3:**
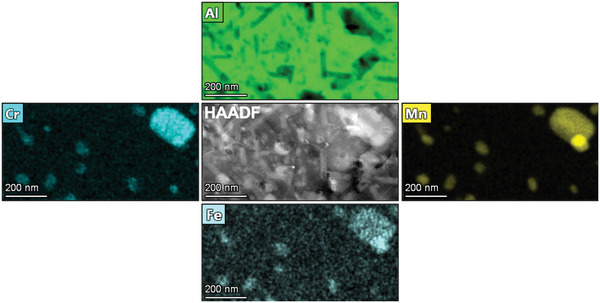
Cr‐,Fe‐, and Mn‐rich particles commonly observed in the pristine microstructure of the crossover AlMgZn alloy. Darker regions in the Al map correspond to the T‐phase precipitates which are analysed in detail in Figure 4.

**Figure 4:**
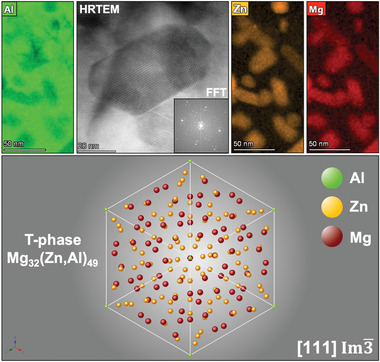
Nanoscale design of the crossover AlMgZn alloy comprises the nucleation, growth and stabilization of an intermetallic hardening phase known as T‐phase with reported stoichiometry of Mg_32_(Zn,Al)_49_. The T‐phase crystal structure herein presented was simulated within the CrystalMaker software using existing reference literature data.^[^ ^75^, ^76^, ^77^, ^78^, ^79^, ^80^, ^83^, ^84^, ^85^ ^]^ The orientation relationship shown in the figure was by Ryum as ${\left(0\bar{1}1\right)}_{\text{T-phase}}\left|\right|{\left(001\right)}_{\text{Al}}$.^[^ ^86^ ^]^

Typical low‐magnification microstructure of the crossover AlMgZn alloy is shown in **Figure** 3. Cr‐, Fe‐ and Mn‐rich particles with sizes between 100–300 nm are observed in the alloy microstructure in its pre‐irradiated condition (i.e. pristine condition). A brighter HAADF signal from such particles is noted when compared with the Al matrix. At higher magnifications, nanometer‐sized precipitates are found to compose the microstructure of the alloy corresponding to the hardening phase for this system. As shown in **Figure** 4, the combination between Mg and Zn in this alloy system gives rise to the precipitation of T‐phase which is of cubic crystal structure with reported stoichiometry of Mg_32_(Zn,Al)_49_.^[^ ^75^, ^76^, ^77^, ^78^, ^79^, ^80^, ^83^, ^84^, ^85^ ^]^ It is worth emphasising that in the EDX maps exhibited in Figure 4, T‐phase precipitates show Mg and Zn enrichment and most of them have sizes within the range from 10 to 200 nm.

A particular aspect of the T‐phase precipitation in the crossover AlMgZn alloy microstructure is that when the pre‐irradiated sample is screened within the TEM along the [001]–FCC Al zone axis, sharp diffraction spots corresponding to the T‐phase can be observed either using fast Fourier transformation (FFT) from HRTEM micrographs or in selected‐area electron diffraction (SAED) patterns.

A FFT generated from a HRTEM micrograph of typical T‐phase precipitates is presented in Figure 4. These distinct spots in‐between the Al lattice can be directly attributed to the crystallographic signal of the T‐phase in the crossover AlMgZn alloy. A recent work by some of the present authors^[^ ^78^ ^]^ has shown that different heat treatments affect T‐phase precipitation in the crossover AlMgZn alloy: in an underaged condition (398 K for 3 h) no T‐phase diffraction spots were observed while in an overaged condition (like in this work) T‐phase diffraction spots were present in the SAED patterns. These observations were supported by STEM‐EDX mapping of the different alloy microstructures. Using the software CrystalMaker,^[^ ^87^ ^]^ a crystallographic model for the T‐phase was generated (as shown in Figure 4) using data available in the literature^[^ ^75^, ^76^, ^77^, ^78^, ^79^, ^80^, ^83^, ^84^, ^85^ ^]^ and the crystallographic signals found were properly indexed in a method reported elsewhere.^[^ ^77^, ^78^ ^]^ The crystallographic data files (.cif) – used both to generate such models and perform crystallographic indexing of the T‐phase in this alloy system – are available on the Mendeley dataset linked with this study (see Section 5).

### 3.2 Heavy Ion Irradiation In Situ Within a TEM

**Figure 5:**
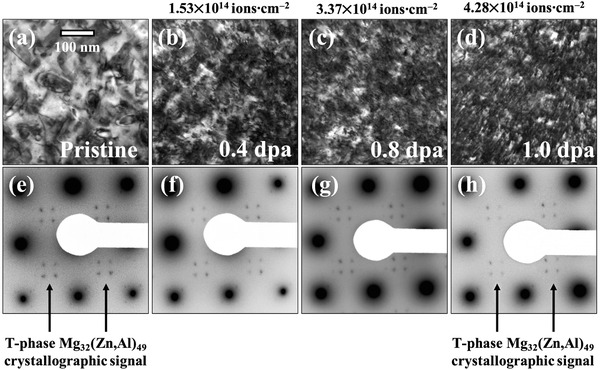
Sequential set of underfocused (1000 nm) BFTEM micrographs and SAED patterns showing the microstructural evolution of the alloy under heavy ion irradiation monitored in situ within a TEM up to a Al‐matrix dose of 1 dpa ($4.28\times 10^{14}\;\mathrm{ions}\cdot {\mathrm{cm}}^{-2}$). Note: the scale bar in (a) applies to all BFTEM micrographs in the figure and the video associated with the results presented in this figure is shared in the Mendeley dataset (see Section 5).

The set of underfocused BFTEM micrographs and SAED patterns (recorded from the same area) showing the evolution of the crossover AlMgZn alloy microstructure under heavy ion irradiation are shown in **Figure** 5. Three important experimental observations can be made based on the obtained results.

Firstly, the crystallographic signal of the T‐phase was used to monitor the evolution of these hardening precipitates as a function of the irradiated dose. As can be observed in the set of SAED patterns in Figures 5e–h, the crystallographic signal of the T‐phase precipitates has not (considerably) changed, neither during nor after irradiation up to a dose of 1 dpa. This is a direct evidence which suggests that the T‐phase precipitates were not subjected to irradiation‐induced dissolution.

Secondly, Cr‐, Fe‐ and Mn‐rich particles were observed to have a different radiation response than the T‐phase precipitates. As observed in the set of BFTEM micrographs in Figure 5a–d, the particles were apparently subjected to irradiation‐assisted dissolution (this observation is also better visualized in the heavy ion irradiation in situ TEM video associated with this manuscript, please see Section 5). It is worth emphasising that no detectable crystallographic signal could be attributed to such particles in the SAED patterns, thus indicating a low volumetric density for such particles (consequently a low diffraction signal in the SAED DPs).

The accumulation of displacement damage is the third notable observation resulting from the heavy ion irradiations in the crossover AlMgZn alloy. This type of radiation damage arises in the form of strong dark contrast in the BFTEM micrographs presented in Figure 5a–d. Such contrast is attributed to the formation of nanometre‐sized black‐spots which are able to evolve to dislocation loops with increasing irradiation dose.^[^ ^88^, ^89^, ^90^ ^]^ At the end of the irradiations—as observed in the BFTEM micrograph in Figure 5d— the alloy matrix is saturated with such displacement damage type defects. It is important emphasising that such displacement damage has been detected within the Al matrix and not within the T‐phase precipitates under the studied conditions.

### 3.3 Alloy Postirradiation Characterization

**Figure 6:**
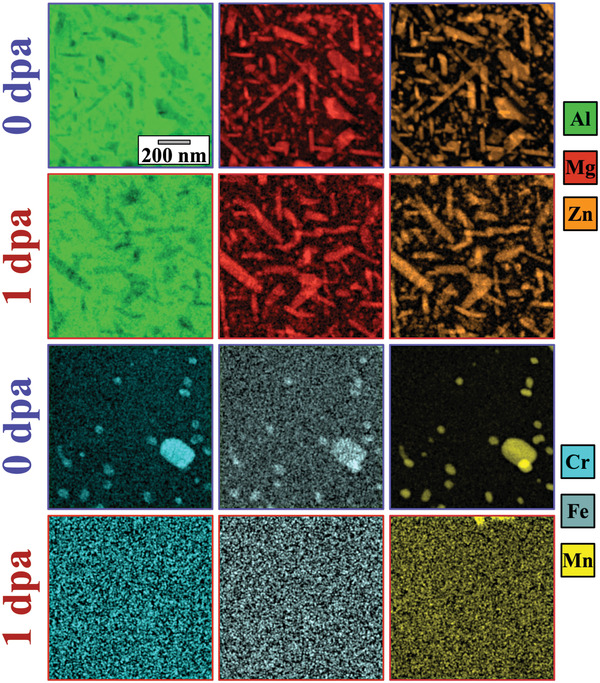
STEM–EDX mapping comparison between the pristine (0 dpa) and irradiated crossover AlMgZn alloy up to 1 dpa (4.28 × 10${}^{14}$ ions·cm${}^{-2}$). Note: the scale bar in the first figure applied to all micrographs.

Elemental mapping of the samples in both pristine and irradiated conditions is exhibited in **Figure** 6. The presence of the T‐phase is noticeable in the pristine alloy and their morphology resemble large plate‐like precipitates. Larger sizes are attained as a consequence of the extensive heat treatment used to process the crossover AlMgZn alloy which is also known as overaged condition (see Section 2.1 ^[^ ^78^ ^]^). In this case study, longer aging times leads to larger hardening phases.^[^ ^91^ ^]^

Interestingly after irradiation, the EDX maps show that the T‐phase did not dissolve, thus corroborating the results presented in Section 3.2 regarding the preservation of the crystallographic signal attributed to the T‐phase in the SAED patterns before, during and after irradiation up to 1 dpa (Figure 5e–h). As opposed to the radiation‐tolerant T‐phase, the Cr‐, Fe‐, Mn‐rich particles dissolved during irradiation.

An important point noticeable in the EDX maps is that the background noise in the Cr, Fe and Mn maps after irradiation appear to be “more cluttered” than the background noise observed in the corresponding elemental maps before irradiation. This suggests that the dissolution of the particles under irradiation may be followed by re‐precipitation into smaller particles. Another important point is that the set of elemental maps from Mg and Zn at 1 dpa shows a slight spherical contrast within and in‐between the long T‐phase precipitates. This indicates that a spherical precipitate – rich in Mg and Zn – has nucleated as a result of the irradiation, but did not grow larger than the pre‐existing ones.

**Figure 7:**
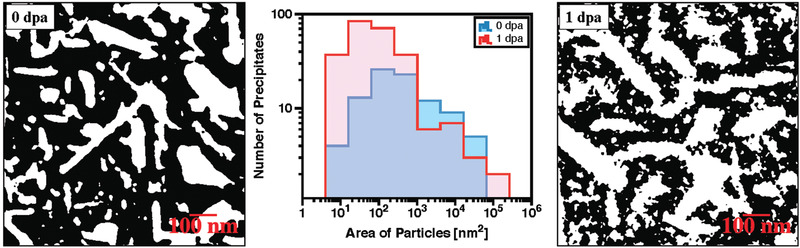
Quantitative size analysis for T‐phase precipitates before (0 dpa) and after irradiation up to 1 dpa ($4.28\times 10^{14}$ $\mathrm{ions}\cdot {\mathrm{cm}}^{-2}$). The micrographs shown in the figure had their threshold levels changed within ImageJ under the same conditions for particle size analysis and they correspond to the EDX maps of the element Mg (from Figure 6) in both pristine and irradiated conditions.

Further analysis of the EDX maps from the T‐phase precipitates was carried out within the software ImageJ. **Figure** 7 shows the result of a particle size analysis performed with the Mg‐signal of the EDX maps shown in Figure 6 before and after irradiation up to 1 dpa. The threshold micrographs (generated under identical conditions) indicate that after irradiation, smaller spherical precipitates are noticeable within the crossover AlMgZn alloy matrix with significant higher areal density when compared with the alloy in its pristine condition. The histogram of area of particles quantitatively confirms such observation: before irradiation, a Gaussian‐like areal profile is observed in contrast with the profile after irradiation, which resembles a log‐normal distribution with the peak shifted to smaller areas, thus indicating the nucleation of smaller precipitates.

## 4 The Radiation Response of the Crossover AlMgZn Overaged Alloy: Critical Discussion

In contrast to the work reported by other authors—Ismail^[^ ^54^ ^]^ using neutrons and Lohmann using protons^[^ ^73^ ^]^ that reported the dissolution of β‐phase precipitates in AlMgSi alloys—intermetallic T‐phase precipitates were identified as the main hardening phase for the developed crossover AlMgZn alloy^[^ ^77^, ^78^ ^]^ and were herein (surprisingly) observed to be radiation resistant to heavy ion irradiation within the dose range analysed. The morphology of the T‐phase precipitates also seems to be unaffected by irradiation, although evidence supporting the occurrence of RIP was presented. RIP manifested by the nucleation of small and spherical Mg‐ and Zn‐rich precipitates within the alloy matrix and at the surfaces of the pre‐existing T‐phases. Such RIP effect may correspond to the formation of new T‐phases precursors or GP zones from the Al–Mg–Zn system. A similar trend was reported by Liu et al.^[^ ^50^ ^]^ with low temperature neutron irradiation in a different AlMgZn alloy: a log‐normal distribution of GP zones was detected after irradiation via X‐ray small angle scattering by estimating both Guinier and Porod radii. In this present work, such a log‐normal distribution was also observed in the crossover AlMgZn alloy after irradiation up to 1 dpa, as shown in the histogram in Figure 7, suggesting that GP zones may form via a RIP process.

Conversely, Cr‐, Fe‐, Mn‐rich particles were observed to dissolve under irradiation. In Al‐based alloys, such particles are often reported as dispersoids with sizes from 10 to 500 nm (or even larger).^[^ ^92^, ^93^ ^]^ Their formation and final size distribution are highly dependent on the homogenization treatment and alloy composition. These particles in Al‐based alloys are known to have high thermodynamic stability and their influence on the alloy is limited to processes related with recrystallization, recovery and grain growth and due to their low volumetric density, they do not promote significant hardening.^[^ ^92^, ^93^, ^94^ ^]^

Dissolution of nanoprecipitates under irradiation is related to ballistic processes such as thermal spikes and large displacement damage cascades.^[^ ^71^, ^72^, ^95^ ^]^ The impact of a heavy ion with a crystal lattice may lead to a highly disturbed area (over the length of the damage cascade) which can raise the local temperature to thousands of Kelvin. This may induce mixing and solid‐state diffusion of (segregated) solutes followed by the destruction/dissolution of the crystal. The irradiation‐assisted diffusion of solutes out of the particles may also induce their reorganisation and re‐precipitation into smaller phases giving the favourable thermodynamic conditions. Although the comparison between the effects of irradiation observed in the particles and the T‐phase suggests that the former is more prone to degrade as a result of the atomic collisions, a more definitive approach to define the reasons behind the radiation tolerance of the latter is a topic of research that remains to be further addressed.

**Figure 8:**
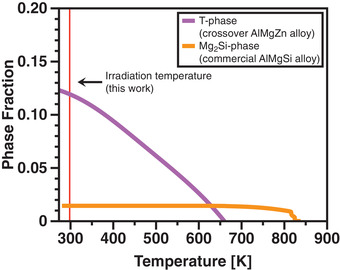
Phase fractions of hardening precipitates in both crossover AlMgZn alloy (T‐phase) and commercial AlMgSi (AA6061) alloy (Mg${}_{2}$Si‐phase) calculated with the Pandat software.^[^ ^96^, ^97^ ^]^ At the irradiation temperature in this work, the T‐phase fraction is one order of magnitude higher than the Mg${}_{2}$Si‐phase fraction.

A particular aspect of the crossover AlMgZn alloy tested in this work may shed light on its interesting irradiation response. As investigated by Lohmann et al.,^[^ ^73^ ^]^ the Mg${}_{2}$Si hardening phase in the AlMgSi alloy (AA6061‐T6) was observed to dissolve completely under irradiation at a dose level of 0.2 dpa while the T‐phase in the crossover AlMgZn alloy did not dissolve up to 1 dpa. Equilibrium thermodynamic calculations of the constitution of both alloys were performed with the Pandat software package^[^ ^96^ ^]^ using the database PanAl2019^[^ ^97^ ^]^ and a major difference between these alloys was found as shown in **Figure** 8. At the temperature of the irradiation experiments performed in this work, the phase fraction of the T‐phase is one order of magnitude higher than the Mg${}_{2}$Si‐phase. This indicates that a higher phase fraction is a desired parameter to design a lightweight stellar‐radiation resistant alloy as it may result in more efficient point defect recombination, leading to radiation damage suppression. The additional formation of T‐phase under irradiation as reported in Figure 7 can be also explained by the fact that its phase fraction is higher at 300 K when compared with the processing temperature at 448 K. The equilibrium thermodynamic calculations in Figure 8 also indicate that T‐phase precipitates are present in the crossover AlMgZn alloy within the temperature range of 273–650 K.

The heavy ion irradiation methodology used in this work is far more aggressive in terms of radiation damage yield than the high‐energy protons from SFs in the stellar environment. A quick SRIM calculation shows that for 1 µm of pure Al, 100 keV Pb ions generate an average of 10${}^{3}$ vacancies per collision while for 2.5 MeV protons, this number is barely 20 vacancies per collision. Thus, the heavy ion irradiation methodology used in this work extrapolate to the conditions found in the solar system either under normal or abnormal conditions. In this way, it is reasonable to assume that the irradiation conditions to which the crossover AlMgZn alloy were subjected in this work is an extreme case scenario. This generation of point defects in excess, results in the accumulation of displacement damage (e.g., black‐spots and dislocation loops) as shown in Figure 5, but voids were not detected during such experiments and up to a dose of 1 dpa. The dislocations can act as nucleation sites for the smaller precipitates that have nucleated (but not grown) as a result of the irradiation which explains the RIP of nanometre‐sized Mg‐, Zn‐rich GP zones and the Cr‐, Fe‐, Mn‐rich re‐precipitation. Obviously, this would embrittle the alloy, but one can predict that in the stellar environment, this displacement damage will be far lower, therefore not causing significant alterations in the crossover AlMgZn alloy microstructure from this point of view.

Mitigation of displacement damage generation could be performed via the design of Al‐based alloys with reduced grain sizes. In its nanocrystalline morphology, the grain boundaries will serve as preferential sinks for radiation‐induced diffusing point defects, thus reducing the yield of black‐spots and dislocation loop formation. This is also a trend observed in the literature of nuclear materials in recent years.^[^ ^98^, ^99^, ^100^ ^]^

Under the extremes of heavy ion irradiation, it is demonstrated that the prototypic alloy studied in this work exhibited a high degree of radiation tolerance and it is a promising candidate for applications in space. This radiation tolerance arises from the fact that the main hardening phase component did not dissolve as a result of the irradiation, in opposition to irradiations of AlMgSi commercial alloys carried out previously by other researchers.^[^ ^54^, ^73^ ^]^

## 5 Conclusions

Historically, space exploration poses a significant challenge to materials scientists and metallurgists. The design of materials that are capable to resist the multiple degradation mechanisms present in the space and that can operate synergistically either under normal or abnormal conditions is a major goal within this industry.

Lightweight materials – such as Al‐based alloys – are required in order to reduce the payload weight and other costs associated with the launch and the whole space program itself. Easy machinability, recyclability, repairability, replaceability combined with high strength are among the qualities which makes Al‐based alloys a strategic asset for the future of space exploration.

Within the solar system, a particular material degradation mechanism is of concern: the radiation damage resulting from the emission of solar energetic particles. Stellar‐radiation is significantly higher when the sun experiences abnormal conditions of weather such as solar flares and coronal mass ejections. Thus, radiation resistance is a key design criterion for new space materials.

In this paper, we investigated the radiation response of a prototypic AlMgZn alloy which has already shown relevant properties for applications in the automotive industry.^[^ ^77^, ^78^ ^]^ The alloy design employs the crossover principle which consists of harnessing the main beneficial properties of two (or more) distinct Al‐based alloy classes, resulting in final alloys with a superior intermix of properties.

It must be emphasized that only the radiation response of the crossover AlMgZn alloy was investigated in this work. As described in Section 1.1, the resistance to multiple degradation mechanisms operating in synergy must be demonstrated prior the utilization of a certain material in the space environment. From the radiation tolerance perspective, one must note that the major conclusions obtained from the experiments performed in this present work may serve only as guidance for the design of a novel class of materials: the stellar‐radiation tolerant and lightweight alloys. Thus, two major guidelines can be summarizzed as follows:

– The hardening phase—responsible to confer strength and structural integrity—must resist irradiation and not dissolve or shrink. Higher phase fractions for hardening precipitates is another desired parameter as this may result in more efficient recombination of excess point defects generated by the atomic collisions. On this direction, the Mg_32_(Zn,Al)_49_ T‐phase showed promising results, although the mechanisms behind its high radiation resistance remains as yet unknown.
– The displacement damage yield must be reduced, i.e., the nucleation and growth of extended radiation‐induced defects (e.g., dislocation loops) must be suppressed in these new alloys as they can increase the hardness by facilitating the radiation‐induced precipitation, possibly resulting in severe embrittlement. Following a recent trend observed in the literature,^[^ ^98^, ^99^, ^100^, ^101^, ^102^ ^]^ new alloys with reduced grain sizes should be designed as the increase in the density of sinks (grain‐boundaries) will result in less accumulation of radiation‐induced point defects within the grains.

Although the underlying physical mechanisms governing the experimentally observed high resistance of the T‐phase to extreme heavy ion irradiation are yet to be clarified, we assume today that for the prototypic alloy design of lightweight alloys for stellar‐radiation environments, a high phase fraction and high complexity are desirable properties of the hardening phase. Additional research is needed for a better understanding of the influence of heat‐treatments on the irradiation response of these new crossover Al‐based alloys, mainly when considering the special intermetallic superstructures as hardening phases (such as the T‐phase) and some of their morphological aspects such as size‐distribution and spacing. Eventual morphological changes on the T‐phase precipitates as well as the dissolution and possible re‐precipitation of Cr‐, Fe‐ and Mn‐rich particles under irradiation should be better evaluated with other experimental characterization methods such as atom probe tomography. Ion irradiation in the bulk crossover AlMgZn alloys followed by mechanical testing and nanoindentation should be also considered as a next step for research.

## Conflict of Interest

The authors declare no conflict of interest.

## Data Availability

The raw and processed data required to reproduce these findings are available to download via the link https://doi.org/10.17632/s5npfgnbv6.1 permanently stored at the Mendeley Data repository.
